# A B73×Palomero Toluqueño mapping population reveals local adaptation in Mexican highland maize

**DOI:** 10.1093/g3journal/jkab447

**Published:** 2022-01-03

**Authors:** Sergio Perez-Limón, Meng Li, G Carolina Cintora-Martinez, M Rocio Aguilar-Rangel, M Nancy Salazar-Vidal, Eric González-Segovia, Karla Blöcher-Juárez, Alejandro Guerrero-Zavala, Benjamin Barrales-Gamez, Jessica Carcaño-Macias, Denise E Costich, Jorge Nieto-Sotelo, Octavio Martinez de la Vega, June Simpson, Matthew B Hufford, Jeffrey Ross-Ibarra, Sherry Flint-Garcia, Luis Diaz-Garcia, Rubén Rellán-Álvarez, Ruairidh J H Sawers

**Affiliations:** 1 Laboratorio Nacional de Genómica para la Biodiversidad/Unidad de Genómica Avanzada, Centro de Investigación y de Estudios Avanzados, Instituto Politécnico Nacional (CINVESTAV-IPN), Irapuato, Guanajuato 36821, México; 2 Department of Plant Science, The Pennsylvania State University, State College, PA 16802, USA; 3 Department of Evolution and Ecology, UC Davis, CA 95616 USA; 4 Department of Botany, University of British Columbia, Vancouver, BC V6T 1Z4, Canada; 5 International Center for Maize and Wheat Improvement (CIMMyT), De México 56237, México; 6 Jardín Botánico, Instituto de Biología, Universidad Nacional Autónoma de México, Ciudad de México 04510, México; 7 Department of Ecology, Evolution, and Organismal Biology, Iowa State University, Ames, IA 50011, USA; 8 Center for Population Biology, and Genome Center, UC Davis, Davis, CA 95616, USA; 9 U.S. Department of Agriculture, Agricultural Research Service Plant Genetics Research Unit, Columbia, MO 65211, USA; 10 Campo Experimental Pabellón-INIFAP. Carretera Aguascalientes-Zacatecas, Aguascalientes, CP 20660, México; 11 Department of Molecular and Structural Biochemistry, North Carolina State University, Raleigh, NC 27695, USA

**Keywords:** maize, local adaptation, highland adaptation, Palomero Toluqueño, tassel branching, macrohairs

## Abstract

Generations of farmer selection in the central Mexican highlands have produced unique maize varieties adapted to the challenges of the local environment. In addition to possessing great agronomic and cultural value, Mexican highland maize represents a good system for the study of local adaptation and acquisition of adaptive phenotypes under cultivation. In this study, we characterize a recombinant inbred line population derived from the B73 reference line and the Mexican highland maize variety Palomero Toluqueño. B73 and Palomero Toluqueño showed classic rank-changing differences in performance between lowland and highland field sites, indicative of local adaptation. Quantitative trait mapping identified genomic regions linked to effects on yield components that were conditionally expressed depending on the environment. For the principal genomic regions associated with ear weight and total kernel number, the Palomero Toluqueño allele conferred an advantage specifically in the highland site, consistent with local adaptation. We identified Palomero Toluqueño alleles associated with expression of characteristic highland traits, including reduced tassel branching, increased sheath pigmentation and the presence of sheath macrohairs. The oligogenic architecture of these three morphological traits supports their role in adaptation, suggesting they have arisen from consistent directional selection acting at distinct points across the genome. We discuss these results in the context of the origin of phenotypic novelty during selection, commenting on the role of *de novo* mutation and the acquisition of adaptive variation by gene flow from endemic wild relatives.

## Introduction

Climatic trends and a need to reduce the level of agronomic inputs have fostered interest in the development of crop varieties that show stable performance in the face of diverse, and potentially unpredictable, environmental challenges. One approach to enhance stress tolerance in the cultivated genepool is to explore diversity at the extremes of a crop’s distribution (Emon et al. 2015; Dwivedi et al. 2016; Corrado and Rao 2017; Sousaraei et al. 2021). Thousands of years of effort and care by the world’s traditional farming communities have generated a rich diversity of landrace varieties, collectively adapted to a far broader ecological range than modern breeding material (Bellon et al. 2018). In addition to being a valuable source of adaptive variation, landraces serve to illustrate the mechanisms whereby plants can adapt to environmental stress.

Strong directional selection imposed by prevailing conditions tends to produce highly specialized forms that perform well in their home environment, but relatively poorer in other locations, a process referred to as *local adaptation*. Local adaptation is defined formally as superior performance of local genotypes in their native environment vs nonlocal genotypes (Mitchell-Olds et al. 2007; Hall et al. 2010; Anderson et al. 2011, 2013). Concomitantly, the average performance of a locally adapted variety over a range of environments may be poorer than that of a generalist that maintains a reasonable level of performance in all environments. Experimentally, the best demonstration of local adaptation is the reciprocal transplant experiment, in which varieties of interest are evaluated in a series of common gardens covering the range of their home environments. Local adaptation can be described in the context of *genotype* × *environment interaction* (GEI), *i.e.* the degree to which the relative performance of a given variety compared with others depends on environmental conditions (Scheiner 1993; Assmann 2013; Juenger 2013; El-Soda et al. 2014).

By definition, all varieties will suffer reduced performance when challenged by environmental stress. GEI describes variety-specific deviations from the environmental main effect: some varieties suffer more than average, while others are better able to mitigate the impact of the stress. In extreme cases, the relative performance of varieties changes between environments, a scenario referred to as *rank changing* GEI. While stress is often considered with respect to a single suboptimal factor, the same framework applies equally to the complex pattern of challenges presented by different localities. It can be seen that rank changing GEI underpins local adaptation, as defined above. With the advent of comparative genomics and greater understanding of the physiology and cell biology of environmental responses, it has become feasible to begin to characterize the genetic basis of local adaptation (Lovell et al. 2021). Two principal modes of gene action have been proposed to drive rank changing GEI, namely *conditional neutrality* and *antagonistic pleiotropy*. Under conditional neutrality, a given genetic variant is linked to phenotypic change in some environments but not others. A complementary suite of conditionally neutral loci would, theoretically, be sufficient to generate rank changing GEI. Under antagonistic pleiotropy, the sign of the effect of a given variant changes between environments, *e.g.* a beneficial allele in one environment becomes deleterious in another, with a behavior at a single variant that directly mirrors the whole genotype pattern of GEI. In either case, GEI allelic effects are relative to the overall environment mean, and an allele might be superior to another in a given environment even if the overall performance is reduced under stressful conditions. In practice, both conditional neutrality and antagonistic pleiotropy will typically contribute to GEI and, indeed, classification of any given variant will be specific to the environments under consideration (Fournier-Level et al. 2011). In addition, distinguishing conditional neutrality from antagonistic pleiotropy may be limited by statistical power in any given design. To date, studies of local adaptation in *Hordeum spontaneum* (Verhoeven et al. 2004, 2008), the annual grass *Avena barbata* (Gardner and Latta 2006; Latta et al. 2007, 2010), the model plant *Arabidopsis thaliana* (Weinig et al. 2003; Fournier-Level et al. 2011), and *Mimulus guttatus* (Lowry et al. 2009; Hall et al. 2010) have predominantly found cases of conditional neutrality. That said, examples of antagonistic pleiotropy do exist, although mostly limited to plant model organisms (Scarcelli et al. 2007; Hall et al. 2010; Todesco et al. 2010; Anderson et al. 2013). An important consequence of the genetic architecture of local adaptation is the degree to which the specialist is constrained by tradeoffs that impose an unavoidable cost of poor performance outside of the home environment. In terms of plant breeding, there are analogous implications with regard to how extensively a given variety can be used and how robust yields will be in the face of changing environmental conditions.

In addition to their intrinsic value, crop landraces provide an excellent system to study local adaptation, especially with regard to rapid change required over a short timeframe. Landraces are dynamic populations, each with a unique identity shaped by biotic and abiotic stresses, crop management, seed handling, and consumer preferences. As such, landraces are the product of both direct and indirect farmer selection, natural selection in response to the local environment and exchange through traditional seed flow networks (Louette et al. 1997; Cleveland and Soleri 2007; Mercer et al. 2008; Mercer and Perales 2019). Typically, they are cultivated under low-input conditions and produce a modest but stable yield (Zeven 1998; Breseghello and Coelho 2013; Dwivedi et al. 2016). The sustained association of a given landrace population with a given locality results in local adaptation, in the same way it is seen in wild populations, demonstrable by reciprocal transplantation (Janzen et al. 2021).

Maize (*Zea mays* ssp. *mays*) was domesticated from Balsas teosinte (*Z.may*s subsp. *parviglumis*; Matsuoka et al. 2002), about 9,000 years ago, in the basin of the Balsas River in Mexico (Piperno et al. 2007). After domestication, maize dispersed and was successfully established in different environments throughout the Americas and, eventually, across the world. In Mexico alone, 59 different native landraces of maize have been described, grown from sea level to 3,400 m.a.s.l., in a range of environments, from semidesert to regions with high humidity and temperature (Ruiz Corral et al. 2008). One of the key events during the early expansion of cultivated maize was the colonization of the central highlands of Mexico. The central Mexican highlands are characterized by low atmospheric pressure and temperature, frequent frosts and freeze–thaw cycles from October through to late March, high UV-B radiation, seasonal precipitation, and low phosphorus availability due to the volcanic origin of the soil (Bellon et al. 2005; Körner 2007; Mercer et al. 2008; Espinosa-Calderón et al. 2011; Galván-Tejada et al. 2014). Previous work has highlighted the impact of low temperature and high light intensity on unadapted maize varieties, which suffer metabolic lesions in chlorophyll synthesis, leading to increased photodamage and chlorophyll turnover (McWilliam and Naylor 1967). Interestingly, these cold stress-induced symptoms were not observed in highland maize. Highland maize varieties have to mature and complete grain filling before the first frosts and the end of the growing season (Alvarado-Beltrán et al. 2019). In warmer lowland conditions, highland material is precocious, flowering in as little as 40–50 days.

In the central Mexican highlands, farmers have adapted their management practices to improve the chances of obtaining a successful harvest (CIMMyT and Bjarnason 1994; Eagles and Lothrop 1994). To maximize the length of the growing season, farmers sow early, before the onset of the annual rains. Traditionally, seeds are deep planted (10–25 cm) to access residual soil moisture and to protect from frost damage. This practice allows varieties that require 160–180 days to reach maturity to be grown in areas with a frost-free season of 90–120 days. The volcanic soils of the Mexican highlands have low pH, restricting the availability of phosphate to the plant (Bayuelo-Jiménez et al. 2011). Although displaying enhanced phosphorus use efficiency (Bayuelo-Jiménez and Ochoa-Cadavid 2014), Mexican highland landraces tend to show restricted root growth (CIMMyT and Bjarnason 1994; Eagles and Lothrop 1994). To compensate for weak root development and prevent lodging, plants may be hilled (piling of soil around the base of the plant) several times during vegetative growth.

**Fig. 1.**
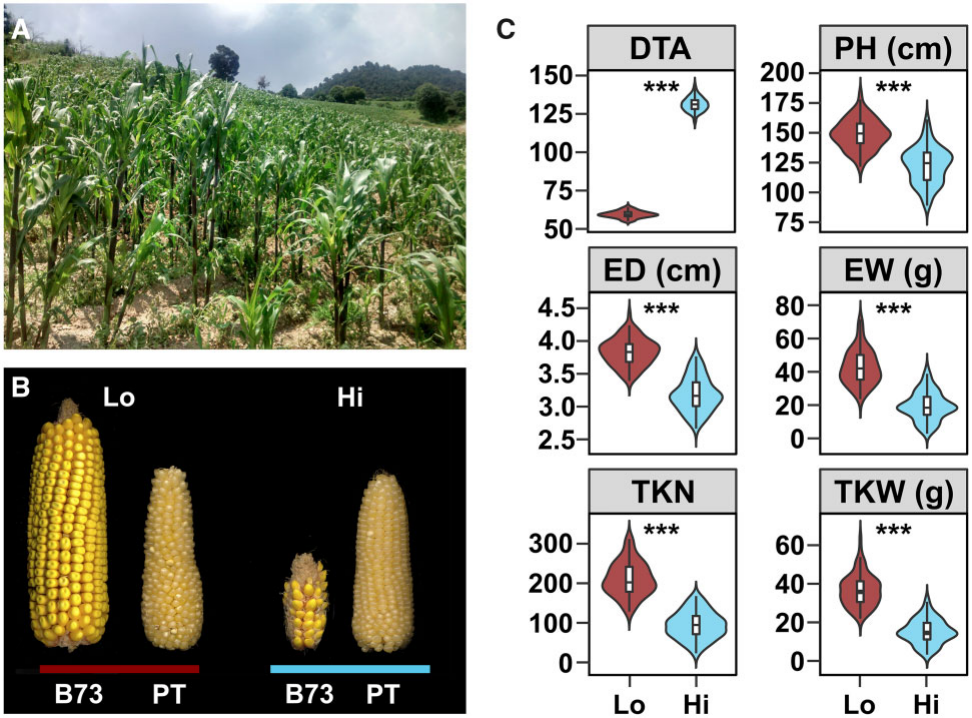
The highland environment impacts maize growth and productivity. a) A highland cultivated maize field at 3,000 m.a.s.l. near the Nevado de Toluca volcano, State of Mexico, Mexico (19.121702, −99.660812). b) Representative ears of the temperate adapted inbred line B73 and the Mexican highland landrace PT grown in lowland (red bar; 54 m.a.s.l.; Valle de Banderas [Lo], Nayarit, Mexico) and highland (blue bar; 2610 m.a.s.l.; Metepec [Hi], State of Mexico, Mexico) field sites. c) Effect of the highland environment on plant performance. Distribution of trait values for 120 B73×PT BC_1_S_5_ lines grown in the lowland (Lo; red) and highland (Hi; blue) field sites. Trait codes: DTA—*days to anthesis* (days); PH—*plant height* (cm); ED—*ear diameter* (cm); EW—*ear weight* (g); TKN—*total kernel number*; TKW—*total kernel weight* (g). Fitted values were derived by adding G and GEI deviations for each line to the field site average. Boxes represent the interquartile range with the horizontal line representing the median and whiskers representing 1.5 times the interquartile ranges. The shape of the violin plot represents probability density. Support for an environmental main effect shown as *P < 0.05; **P < 0.01; ***P < 0.001

Palomero Toluqueño (PT) is a traditional popcorn distributed in the Mexican highlands, notably in the valley of Toluca, at elevations from ∼2,100 to ∼2,900 m.a.s.l. (Fig. 1a; Wellhausen et al. 1952; Ruiz Corral et al. 2008; Perales and Golicher 2014). Although present day cultivation is limited (https://www.biodiversidad.gob.mx/diversidad/proyectoMaices), PT is considered ancestral to the broader Mexican highland maize group and a progenitor of more productive modern highland landraces (Reif et al. 2006; Arteaga et al. 2016). PT has a relatively small genome and was selected as the target of the first landrace maize genome sequencing study (Vega-Arreguín et al. 2009; Vielle-Calzada et al. 2009). Subsequent work has continued to explore gene expression variation in PT (Aguilar-Rangel et al. 2017; Crow et al. 2020) and characterize wild-relative introgression through comparative genomic analysis (Gonzalez-Segovia et al. 2019). In contrast, B73 is an inbred line developed in the 1970s by Iowa State University as part of the US maize corn belt breeding program. B73 has good combining ability and became a key ancestral female line in hybrid breeding programs (Troyer 1999; Iowa State University 2009). B73 was selected for the first genome assembly in maize (Schnable et al. 2009) and remains as the primary reference genome (Jiao et al. 2017).

In this work, we characterize a mapping population generated from the cross of B73 and PT. We demonstrate local adaptation in PT and characterize associated genetic architecture by evaluation of our mapping population in lowland and highland field sites. We identified quantitative trait loci (QTL) linked to phenology, morphology, and yield components, including evidence of QTL× environment interaction (QEI). Overall, morphological QTL were stable across environments with either little QEI or mild scaling effects. We observed stronger QEI associated with yield components. We found evidence for relatively complex genetic architectures associated with putatively adaptive morphological traits. We discuss the implications of these results with respect to the origin of adaptive variation during rapid local adaptation in cultivated species.

## Materials and methods

### Plant material

To generate a biparental mapping population, an F_1_ was generated from the cross between the reference inbred line B73 and pollen pooled from several individuals of PT, an open pollinated landrace endemic of the Mexican highlands. The PT accession used was CIMMYTMA 2233, MEXI 5 (https://doi.org/10.18730/GAKPV) obtained from the International Center for Maize and Wheat Improvement (CIMMyT) seed bank, originally collected near the city of Toluca, Mexico State (19.286184N, −99.570871W) at 2597 m.a.s.l. A single B73×PT F_1_ individual was crossed as male to multiple B73 ears to generate a large BC_1_ population, capturing a single haplotype of PT. The BC_1_ was then self-pollinated for 5 generations from ear-to-row to produce 120 BC_1_S_5_ families, giving a theoretical average of 25% PT genome content to 75% of B73 per family. The same initial crossing strategy was used to generate material from the cross between B73 and the open-pollinated Conico/Celaya accession CIMMYTMA 1872, MICH 21 (https://doi.org/10.18730/GA8DU). B73×Mi21 stocks were further backcrossed to B73 with phenotypic selection for sheath pubescence to produce a segregating BC_5_S_1_ stock. The progenitor B73×PT and B73×Mi21 F_1_ individuals described here are the same as those used in a previous report to derive introgression stocks segregating the *Inv4m* inversion polymorphism (Crow et al. 2020).

### DNA preparation and genotyping

Fifty micrograms of leaf tissue for 100 BC_1_S_5_ families were harvested for each plant in a 2.0-ml tube and then frozen to −80°C. The frozen tissue was ground in a Qiagen TissueLyser II (Cat. ID: 85300) with a 30-Hz frequency for 30 s. After grinding, 300 μl of UEB1 (250 mM NaCl, 200 mM Tris pH 7.5, 25 mM EDTA, 0.5% SDS) buffer were added and the solution was mixed in a Thermomixer at 38°C for 10 min. Two microliters of PureLink RNAse were added and the mix was left incubating for 30 min. After incubation, samples were separated by centrifugation at 14,000 rpm for 10 min at room temperature. Two hundred and fifty microliters of supernatant were recovered and collected in a 1.5-ml tube. Forty microliters of 3M sodium acetate, pH 5.2, and 450 μl of isopropanol were added per tube, and samples incubated for 20 min at 4°C. A further centrifugation step was performed (14,000 rpm, 10 min, room temperature) and the supernatant was discarded. Pellets were washed 2 times with 250 μl of 70% ethanol. The supernatant was discarded, and the pellet was left to dry for 30 min. When the pellet was dry, it was resuspended in 100 μl of milliQ water. DNA was quantified by spectroscopy and adjusted to a concentration of 20 ng/μl. DNA was analyzed by DArTSeq (Edet et al. 2018) at the SAGA (Servicio de Análisis Genético para la Agricultura, https://seedsofdiscovery.org/about/genotyping-platform/) laboratory in CIMMyT, generating ∼30,000 reads per sample.

### Processing of short-read genotyping data and construction of the genetic map

Short-read DNA sequences generated by DArT-Seq were aligned to the v4 B73 reference genome (Jiao et al. 2017) using seqmap (Jiang and Wong 2008). Sequences that aligned to more than 1 physical position in the reference genome or that did not align were discarded. Single nucleotide polymorphism (SNP) calling was performed with TASSEL 5 (Bradbury et al. 2007). SNP calls were transformed to an ABH format: A assigned to B73, B to PT and H to heterozygotes. Sites for which the parental genotype was missing, ambiguous or heterozygous were removed. Two BC_1_S_5_ families with more than 30% of missing SNP information were removed, leaving a total of 98 BC_1_S_5_ families used to estimate the genetic map. SNP calls were processed using genotype-corrector (Miao et al. 2018), which considerably increased the contiguity of haplotypes among chromosomes. A set of 2,067 polymorphic markers were selected for downstream use. The ABH genotype file was visualized using R/ABHgenotypeR (Reuscher and Furuta 2016). Linked markers with shared patterns of segregation were identified with findDupMarkers function of R/qtl package (Broman et al. 2003). Removing redundant makers reduced the final set to 918 polymorphic markers. The marker order was anchored according to their physical position in the v4 B73 reference genome and the genetic distance was estimated using R/ASmap::mstmap (Taylor and Butler 2017) under the Kosambi map function and parameter anchor = T. The resulting map consisted of 98 individuals, 918 markers, and a total length of 654.5 cM (Supplementary Fig. 1). Five individuals from a B73×Mi21 BC_5_S_1_ family segregating sheath pubescence were genotyped using the same DArT-Seq platform as part of a project described previously (Gonzalez-Segovia et al. 2019).

### Field evaluation

The 120 families of the BC_1_S_5_ population were evaluated in the highlands during 2015, 2016, 2018, and 2019 at 2,610 m.a.s.l. in Metepec, Mexico State (Hi. Mean average temperature: 12.4°C; mean annual precipitation: 809 mm; Andosolic soil), and in the lowlands during 2015 and 2016 at 54 m.a.s.l. in Valle de Banderas, Nayarit (Lo. Mean average temperature: 25.8°C; mean annual precipitation: 1,173 mm; Regosolic soil; Supplementary Table 1). BC_1_S_5_ families were evaluated in single-row 15 plant plots in 3 randomized complete blocks in Metepec and 2 blocks in Valle de Banderas. B73 and PT parents were inserted randomly in each block during 2015 and 2016. Weeds and insects were controlled by chemical methods as needed. The Valle de Banderas field site was provided with a ferti-irrigation system. The Metepec site was rain-fed with supplemental sprinkler irrigation after planting as needed. Sixteen phenotypic traits were measured (Table 1). Traits were evaluated as follows: *days to anthesis* (DTA) as the number of days from planting to when 50% of the plants in the row were shedding pollen; *days to silking* (DTS) as the number of days from planting to when 50% of the plants in the row had visible silks; *anthesis-silking interval* (ASI) as the difference between male and female flowering; *plant height* measured from the base of the plant to the to the base of the tassel; *ear height* (EH) measured from the base of the plant to the first ear node; *tassel branch number* as the number of primary branches in the tassel; *tassel length* (TL) as the distance from the base to the tip of the tassel; *ear weight* (EW) as the weight of the whole dried ear, including cob and grain; *ear length* measured from the base to the apex of the ear; *ear diameter* (ED) measured at the middle of the ear; *kernel row number* counted at the middle of the ear; *kernels per row* counted from the base to the apex of the ear; *total kernel weight* (TKW) as the weight of all dried grain shelled from the ear and weighed without the cob; *anthocyanin pigment intensity* (INT) was evaluated at the internode immediate below the highest ear of the plant on a semiquantitative scale ranging from 0 (completely green internode) to 4 (intense red or brown coloration); sheath pubescence was evaluated as a *hair score* calculated from 2 semiquantitative measures taken at the third internode from the top of the plant: macrohair density ranging from 0 (glabrous) to 4 (very hairy), and macrohair pattern, the distribution of the macrohairs on the internode, from 0 (glabrous sheath), 1 (marginal hairs), 2 (heterogeneous sheath pubescence) to 3 (uniform sheath pubescence). Plants with pattern 0 or 1 where given a hair score of 0 for absence of the medial sheath pubescence characteristic of Mexican highland maize. Plants with pattern 2 or 3 where given the density value as the hair score.

**Table 1.** Description of phenotypic traits.

Trait	Description	Unit	Mean Lo	Mean Hi	*H* ^2^ (%)	E	G	**G** ×**E**
DTA	Days to anthesis	d	60	131	52.2	^c^	^c^	^c^
DTS	Days to silking	d	60	131	43.3	^c^	^a^	^c^
ASI	Anthesis-silking interval	d	0.30	0.46	18.2			
PH	Plant height	cm	149	123	76.7		^c^	^c^
TBN	Tassel branch number	Count	5	4	—		—	—
EW	Ear weight	g	42.69	19.60	22.0	^b^		^c^
EL	Ear length	cm	8.8	7.3	52.6		^c^	^c^
ED	Ear diameter	cm	3.8	3.2	51.3	^b^	^b^	^c^
EH	Ear height	cm	65.3	49.7	70.8	^a^	^c^	^b^
KRN	Number of kernel rows	Count	17	14	—	^a^	—	—
KPR	Number of kernels per row	Count	20	13	—	^a^	—	—
TKW	Total kernel weight	g	36.50	15.31	25.1	^b^		^c^
TKN	Total kernel number	Count	210	94	25.3	^b^		^c^
TL	Tassel length	mm	28.3	21.6	75.2		^c^	^b^
INT	Pigment intensity	Score	0	2	—		—	—
MH	Sheath macrohairs	Score	0	0	—	—	—	—

Mean values for lowland (Lo) and highland (Hi) sites. *H*^2^, broad-sense heritability from mixed models. Significance of environment (E), genotype (G), and genotype by environment (G×E) model terms are given as:

a *P* < 0.05;

b *P* < 0.01;

c *P* < 0.001; blank: *P* > 0.05; —: not estimated.

### Data preparation and trait estimation

Preparation of trait data and QTL mapping were performed using R Statistics (R Core Team 2019). Data collected from each single-row plot were collapsed to a single value per plot: plot medians were taken for traits scored on multiple individuals; plot level traits such as flowering time were unchanged. Data were trimmed to remove outliers per trait/location (Hi or Lo) using R/graphics::boxplot default criteria. Continuous traits [ASI, DTA, DTS, ED, EH, EL, EW, PH, *total kernel number* (TKN), TKW, and TL] were further adjusted on a per block basis to a spline fitted using R/stats::smooth.spline against row number to reduce spatial variation at the subblock scale. Spline fitting was not applied to any block containing less than 50 plots. The final dataset contained 4 years of data for location Hi (123 genotypes from 1 block in 2015, 105 genotypes from 3 blocks in 2016, 140 genotypes from 2 blocks in 2018, and 110 genotypes from 1 block in 2019), and 2 years of data for location Lo (123 genotypes from 1 block in 2015, and 117 genotypes from 2 blocks in 2016). For each continuous phenotypic trait, a mixed linear model was fitted using restricted maximum-likelihood with R/lme4::lmer. To fit the model, a location-year variable was generated to represent location by year combinations, and a location-year-block variable was generated to represent location, year, and block combinations, such that:

$$y_{\mathrm{ijkm}}=\mu +E_{i}+G_{j}+GE_{ij}+Y_{k}+B_{m}+\varepsilon_{\mathrm{ijkm}}$$

where the response variable $y_{\mathrm{ijkm}}$ is a function of the overall mean (μ), fixed effect of location ${(E}_{i})$, random effect of genotype $\left(G_{j}\right)$, genotype by location interaction $\left(GE_{ij}\right)$, location-year term ($Y_{k}$), location-year-block term ($B_{m})$, and the residual. BLUP values for the genotypic effect (G) and genotype by location interactions (GEI) were extracted using R/lme4::ranef. We calculated BLUP values for each genotype and location combination (G + GEI) by adding genotype BLUPs and GEI BLUPs (Olivoto et al. 2019). We also calculated fitted values by adding BLUPs to the appropriate means for data visualization and downstream analyses using natural units: for the genotype main effect, fitted values were calculated by adding genotype BLUPs to the grand mean; fitted values for each genotype and location combination were calculated by adding G + GEI BLUPs to the location mean. The significance of the environment, genotype, and genotype by environment effects were evaluated by comparing the full model and the reduced model using the likelihood ratio test for continuous phenotypic traits. For phenotypic traits with count and scale data, 2-group Wilcoxon tests were conducted to evaluate the difference between the two locations. Broad-sense heritability for each continuous trait was estimated based on the linear mixed model results (Holland et al. 2003).

### QTL mapping

The BLUPs for continuous traits, the medians for count traits and the mode for semiquantitative scale traits were used as phenotypic inputs for QTL mapping. Phenotypic scores were selected/combined to perform four distinct analyses: (1) GEN: the genotype main effect (G) of the mixed linear model for continuous traits and the median/mode across all plots for other traits; (2) Lo: G + G×E term for Lo for continuous traits and the Lo median/mode for other traits; (3) Hi: G + G×E term for Hi for continuous traits and the Hi median/mode for other traits; (4) GEI: the difference between Hi and Lo GEI BLUPs for continuous traits and the difference between Hi and Lo median/mode for other traits.

Individual QTL were detected using single QTL scan and multiple QTL mapping (MQM) with R/qtl::scanone (default options; Haley–Knott regression; Broman et al. 2003) and R/qtl::MQM (default options; 100 autocofactors, step.size = 1, window.size = 25; Arends et al. 2010), respectively. Genome-wide LOD significance thresholds were established at α = 0.05 by 1,000 permutations of scanone and MQM models. Individual QTL were combined in an additive multi-QTL model with R/qtl::makeqtl and their positions refined with R/qtl::refineqtl. The function R/qtl::addqtl was used to detect additional QTLs in a multiQTL context with a LOD threshold of 3 for inclusion. Thresholds added to multiQTL LOD plots were calculated assuming 2ln10 ×LOD to follow a *χ*^2^ distribution with 1 degree of freedom (Broman et al. 2003). The final multi-QTL model was applied using R/qtl::fitqtl (Haley–Knott regression) to obtain the refined position and variance contribution. Significance levels of the full model and the component QTL terms were obtained from the drop-1 ANOVA table. Bayes confidence intervals were obtained from the fitqtl model. The effect size and effect plots of each individual term of the full model were obtained with R/qtl::effectplot. The effects of the B73 and PT alleles in Lo and Hi environments were visualized as the standardized G + G× E median value of all families in each genotype: site class. To directly compare these effects, ANOVA and a post hoc Tukey HSD tests were performed. Pairwise comparison of the number of families with each hair score in different qMH genotypic classes was performed with R/stats::fisher.test.

### Environmental genome-wide association analysis

An environmental genome-wide association analysis study (eGWAS) was performed to measure the association between genetic variation and the elevation of native environment for landrace accessions across Mexico, as previously described (Gates et al. 2019). The dataset consisted of 1,830 Mexican maize landrace accessions from the CIMMyT Maize Germplasm Bank with elevation data, genotyped for 440,000 SNPs (Romero Navarro et al. 2017; Gates et al. 2019). We used a linear model to fit the genotypic data and elevation as previously described (Gates et al. 2019). The first five eigenvectors of the genetic relationship matrix were included in the linear model to control for the population structure. The top 1,000 SNPs with the strongest association with elevation were selected and used in downstream analysis.

## Results

### The stress of the highland environment limits maize growth and productivity

To characterize the genetic architecture of highland adaptation in Mexican native maize (Fig. 1a), we crossed the highland landrace PT to the US reference inbred line B73 and derived 120 BC_1_S_5_ families. When generating the BC_1_, we used a single F_1_ individual as a male to pollinate several B73 females, ensuring that a single PT haplotype was captured from the open-pollinated donor accession. As a consequence our mapping population was bialleleic, *i.e.* segregating for B73 and a single PT allele at any given locus. BC_1_S_5_ families were genotyped using the DArT Seq platform (http://www.diversityarrays.com/) and a final set of 918 markers and 98 BC_1_S_5_ families were used for QTL mapping. On average, the BC_1_S_5_ families conformed to the expectation of ∼25% PT genome in a B73 background, with homozygosity >98% (Supplementary Fig. 1).

We evaluated the B73 ×PT BC_1_S_5_ families and parents in Mexican lowland (Valle de Banderas, Nayarit at 54 m.a.s.l.) and highland (Metepec, Mexico State, at 2,610 m.a.s.l.) field sites over several seasons. Lowland trials were conducted during the dry season from November to March with supplemental irrigation. Highland trials were conducted in a rain-fed field in the standard Highland cycle from April to November. We collected data on a range of phenological, morphological and agronomic traits (Table 1). We used a mixed linear model to extract environmental and genetic main effects and GEI effects for each trait. B73 and PT parents showed a classic pattern of rank-changing GEI for yield components across the two locations, demonstrating adaptation of PT to the highland environment (Fig. 1b and Supplementary Fig. 2). The negative impact of the highland environment on B73 was dramatic, while PT was more stable across the two sites. Across the BC_1_S_5_ population, there was a significant environmental effect on 9 of 16 traits (Fig. 1c, Supplementary Fig. 3, and Table 1). Average flowering (*days to anthesis* and *days to silking*), measured in chronological days, was delayed in the highland site by 71 days. Overall, plants in the highlands were shorter in stature (*plant height*, *ear height*) and produced smaller ears (*ear diameter*, *ear weight*) bearing fewer grains (*total kernel number*) (Fig. 1c and Table 1). Average *total kernel weight* per plant dropped from 36.5 to 15.3 g, a reduction of 58%, from the lowland to highland field (Fig. 1c and Table 1).

### Segregation in the BC_1_S_5_ reflects GEI seen in the B73 and PT parents

**Fig. 2.**
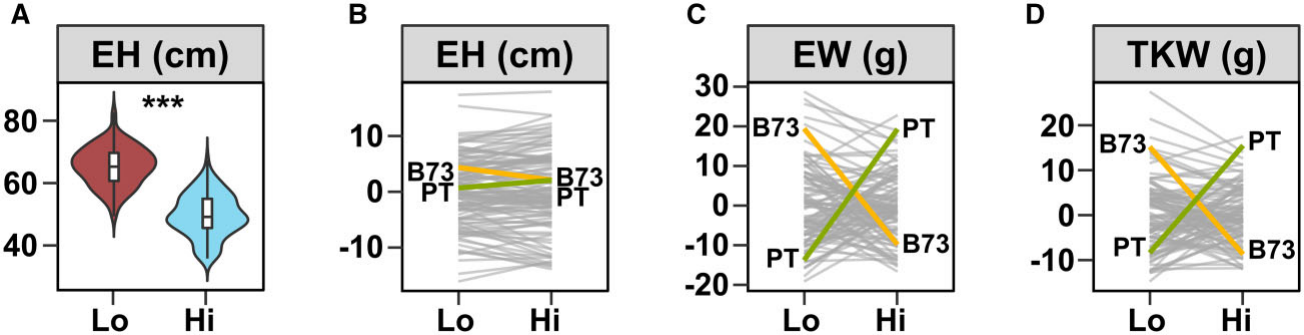
Extensive GEI was observed for yield components. a) Distribution of *ear height* (EH) in lowland (Lo) and highland (Hi) field sites. Boxes represent the interquartile range with the horizontal line representing the median and whiskers representing 1.5 times the interquartile range. The shape of the violin plot represents probability density. b) Reaction norm plot for EH, showing little GEI. Values shown are G + GEI deviations from the field site average. Line segments connect values for each RIL genotype in the 2 field sites. B73 (yellow) and PT (green) parental values are shown; c) and d) as b), showing extensive rank-changing GEI associated with *ear weight* (EW) and *total kernel weight* (TKW), respectively. Support for GEI shown as *P < 0.05; **P < 0.01; ***P < 0.001

Having characterized the main effect of the highland environment, we explored GEI among the BC_1_S_5_ families. For certain traits, such as *ear hieght*, there was a significant GEI effect, but limited rank-changing among the lines (Fig. 2, a and b, Supplementary Fig. 3, and Table1). In contrast, yield components such as *ear weight* and *total kernel weight* showed extensive rank-changing GEI among BC_1_S_5_ families (Figs. 1c and 2, c and d, Supplementary Fig. 3, and Table 1). Taking *ear weight* as our primary proxy for yield, many BC_1_S_5_ families were more stable with respect to location than the 2 parents, although the majority were inferior to the better parent in either site. That said, we did observe a small number of families that performed well in both locations (Fig. 2c).

### QEI associated with yield components indicates local adaptation at the locus level

**Fig. 3.**
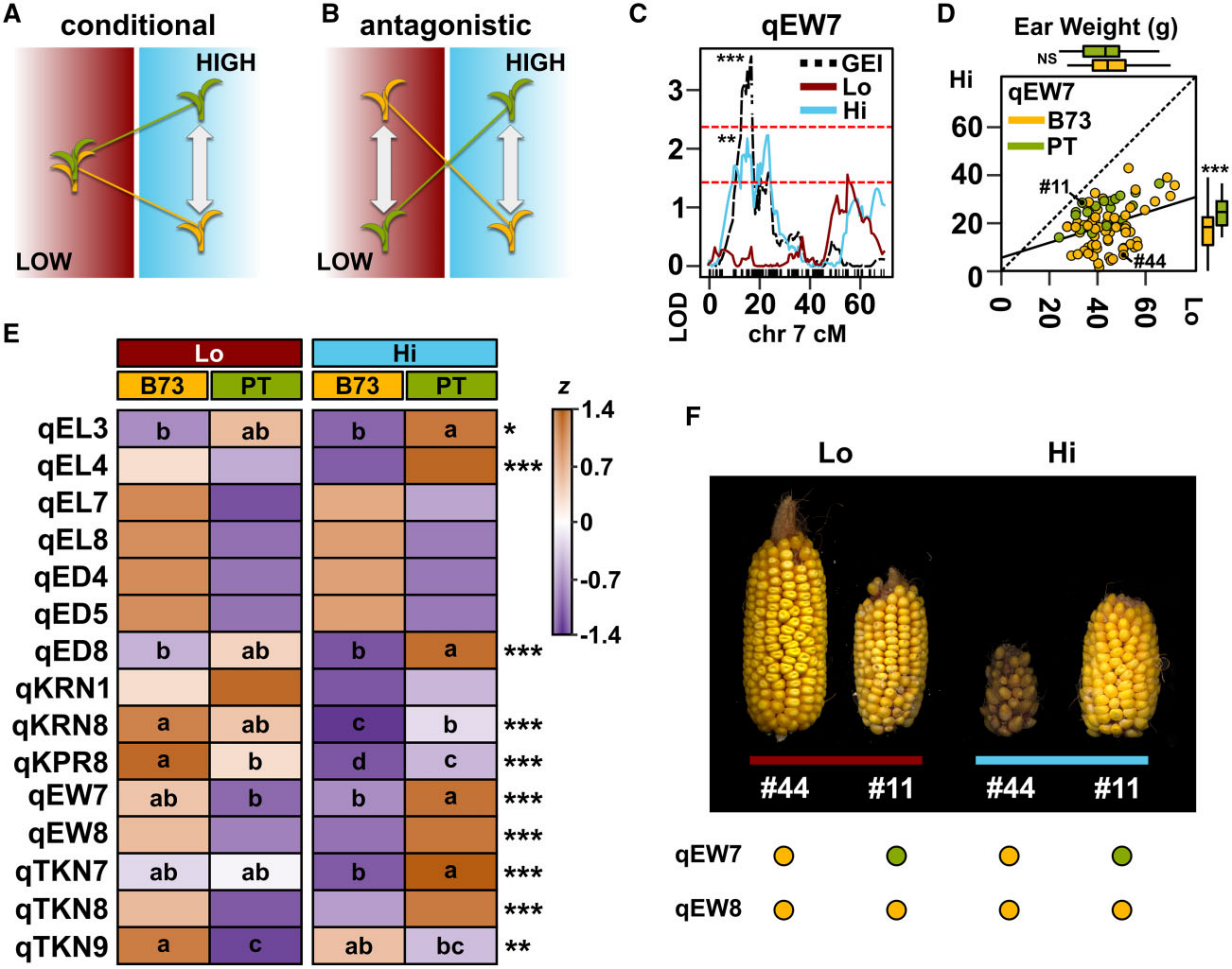
QEIs mirror local adaptation. Schematic of QEI showing a) a *conditional* effect expressed in one environment but not another and b) *antagonistic pleiotropy* in which there is a change in the sign of the QTL effect between environments. c) Heatmap representation of the standardized median G + GEI value for all families with a given genotype (B73 or PT) in lowland (Lo) and highland (Hi) sites, for the named ear morphology and yield component QTL (see Table 2). Asterisks indicate significance in the GEI QTL analysis (*P < 0.05; **P < 0.01; ***P < 0.001). Lowercase letters indicate Tukey means groups from *post hoc* tests applied to all identified QTL. d) LOD support for the *ear weight* QTL qEW7 on the short arm of chromosome (chr) 7. The QTL is well supported by data from the Hi site (blue trace) but not the Lo site (red trace) and is captured by a multiQTL model for GEI (black trace). Upper and lower dashed red lines show theoretical thresholds for inclusion in the multiQTL model at α = 0.001 or 0.01, respectively. e) Scatter plot of EW in Hi against Lo fields. Each RIL is represented by a single point, colored by genotype at qEW7 (yellow, B73; green, PT). RILs shown in f) are labeled. The solid line shows a linear fit through all points. Box plots parallel to the vertical and horizontal axes show the distribution by genotype in Hi and Lo fields, respectively. Boxes represent the interquartile range with the horizontal line representing the median, and whiskers extending 1.5 times the interquartile range. f) Ears of RILs LANMLR17B044 (#44) and LANMLR17B011 (#11) produced in lowland (red bar) and highland (blue bar) fields, showing marked differences in stability with respect to field. Points below the panel indicate QTL genotype.

Extensive GEI for yield components is consistent with complementary conditional effects across several QTL and antagonistic pleiotropy at individual QTL (Fig. 3, a and b). To explore the genetic architecture underlying GEI for yield components in our BC_1_S_5_ families, we performed 4 distinct QTL analyses: separate analyses for highland (Hi) and lowland (Lo) fitted values, an analysis for the genotype main effect (Gen) and an analysis for the GEI effects. Across the 4 analyses, we identified 44 distinct QTLs (Table 2; Supplemental Information). A QTL for a specific trait was considered shared between different analyses if the peak position was on the same chromosome and support intervals overlapped. The GEI analysis alone identified 18 QTL (Table 2). We identified a total of 15 QTL for ear morphology and yield component traits across the 4 analyses (Fig. 3c and Table 2). QTL qEW7 and qEW8 (here and below, we report QTL by trait abbreviation as given in Table 1 and chromosome number) linked to *earweight*, our proxy for yield, were both detected in the GEI QTL analysis (Table 2). For both qEW7 and qEW8, the estimated allele effect was higher for B73 than PT in the lowlands, but higher for PT than B73 in the highlands (Fig. 3, c–e, Mean G + GEI BLUP ±SE: Lo, qEW7 B73 = 1.82 ± 1.16, qEW7 PT = −2.36 ± 2.05; Hi qEW7 B73 = −1.52 ± 0.92, qEW7 PT = 4.01 ± 1.67; Lo, qEW8 B73 = 1.78 ± 1.21, qEW8 PT = −1.48 ± 1.86; Hi qEW8 B73 = −1.74 ± 0.96, qEW8 PT = 3.38 ± 1.48; Supplemental Information). Statistical support, however, was strongest in the highlands for both loci, and we would consider qEW7 and qEW8 as conditional QTL. The qEW7 effect, but not that of qEW8, was recovered in an analysis of variance (ANOVA) applied directly to the standardized effects for the 4 different allele/location groups. A *post hoc* Tukey HSD test supported the conditional benefit of the PT allele at qEW7 in the highlands but did not separate the allele classes in the lowlands (Fig. 3c). Furthermore, qEW7 was supported in the Hi QTL analysis at an α = 0.01, while there was little signal associated with this position in the Lo analysis (Fig. 3d). EW in families carrying the PT allele at qEW7 was more stable between locations than in families carrying the B73 allele, PT families tending to fall above the fitted line of a regression of highland on lowland values, across all families (Fig. 3e). The outlying family LANMLR17B011 carried the PT allele at qEW7 and approached equivalent EW in both field sites (Fig. 3, e and f). In contrast, a family such as LANMLR17B044 carried B73 alleles at both qEW7 and qEW8 and demonstrated better than average *ear weight* in the lowland site but was exceptionally poor in the highlands (Fig. 3, e and f).

**Table 2.** QTLs detected in the B73×PT BC_1_S_5_ RIL population.

QTL	Marker	Chr	PosG (cM)	PosP (Mb)	Interval (Mb)	Var (%)	Analysis
qASI1	1_53029437	1	30.46	53.03	35.2–292.18	10.13	Lo
qASI2	2_241675850	2	77.91	241.68	240.52–244.41	10.88–14.54	Gen, Lo
qASI3	3_229390098	3	65.63	229.39	11.85–234.87	7.29	Gen
qASI8	8_135484500	8	24	135.48	134.32–164.79	15.96–18	Gen, Lo, Hi
qDTA1	1_286172395	1	94.35	286.17	2.81–306.46	6.07–10.19	Lo, GEI
qDTA6	6_166664744	6	22	166.66	165.39–168.82	7.5–13.53	Gen, Hi, GEI
qDTA7	7_165928844	7	48.37	165.93	0.84–173.75	5.51	Hi
qDTA8a	8_21391040	8	0	21.39	25.12–97.57	9.45	Hi
qDTA8b	8_153580487	8	27.5	153.58	148.95–161.29	9.67–21.23	Gen, Lo, Hi, GEI
qDTS1	1_12171293	1	12.47	12.17	2.81–306.46	5.18	GEI
qDTS6	6_166506716	6	23	166.51	165.39–168.12	7.59–15.18	Gen, Hi, GEI
qDTS7	7_163657186	7	48	163.66	15.91–169.56	8.16	Hi
qDTS8	8_161289005	8	31	161.29	133.04–169.05	11.17–16.07	Gen, Hi, GEI
qED4	4_179539232	4	41.65	179.54	163.36–196.26	14.69–14.96	Gen, Lo
qED5	5_190812247	5	31.54	190.81	1.48–198.95	9.2	Lo
qED8	8_97570666	8	7.81	97.57	21.39–112.91	14.86	GEI
qEH1	1_177987239	1	53.68	177.99	162.88–198.3	11.87–14.99	Gen, Hi
qEH7	7_135399817	7	33.26	135.4	131.23–144.37	14.94–16.76	Gen, Lo, Hi
qEL3	3_190627575	3	43.33	190.63	11.5–234.87	13.39	Hi
qEL4	4_74666833	4	23.02	74.67	18.12–161.42	12.34	GEI
qEL7	7_172341845	7	55	172.34	166.94–181.12	12.16	Lo
qEL8	8_175513459	8	43.5	175.51	172.04–179.51	9.25–14.81	Gen, Lo, GEI
qEW7	7_20110508	7	16.9	20.11	9.14–28.5	14.33	GEI
qEW8	8_110436593	8	13.45	110.44	88.36–170.14	11.6	GEI
qMH3	3_156124621	3	28.83	156.12	11.85–161.43	9.43–11.07	Gen, Lo
qMH7	7_155328976	7	43.1	155.33	152.38–164.52	14.21–17.8	Gen, Hi
qMH8	8_122414801	8	18.71	122.41	115.01–123.81	15.42–17.91	Lo, GEI
qMH9	9_65716548	9	12.5	65.72	20.72–124.18	16.61–19	Gen, Lo
qKPR8	8_135484500	8	24	135.48	115.01–164.79	18.86	GEI
qKRN1	1_161556632	1	49.5	161.56	8.68–296.87	12.63	Lo
qKRN8	8_133038186	8	23.42	133.04	82.76–164.79	16.39	GEI
qINT2	2_19456739	2	25.61	19.46	18.31–25.87	22.12–40.03	Gen, Lo, Hi
qINT10	10_117963995	10	19.49	117.96	116.16–138.71	9.47–13.37	Gen, Lo, Hi
qPH1a	1_197051908	1	56.5	197.05	176–199.71	18.88–20.43	Gen, Lo, Hi
qPH1b	1_293830327	1	100.13	293.83	283.08–299.44	12.26	Lo
qPH8	8_149541560	8	27.58	149.54	142.92–164.79	13.69	Lo
qPH10	10_136883639	10	26	136.88	10.27–143.76	11.17	GEI
qTBN2	2_141142660	2	40.88	141.14	70.9–184.93	13.24	Gen
qTBN7	7_121548061	7	24.52	121.55	112.71–121.69	15.86–33.17	Gen, Lo, GEI
qTKN7	7_15912522	7	15.35	15.91	9.14–121.69	17.09	Hi
qTKN8	8_126886782	8	20.81	126.89	82.76–170.04	12.86	GEI
qTKN9	9_124180939	9	19.6	124.18	111.77–135.89	16.39	Lo
qTL1	1_227715124	1	72.51	227.72	224.77–244.86	19.73	GEI
qTL2	2_230998297	2	70.19	231	0.94–241.68	11.56–11.62	Gen, Hi

QTL are named by trait and chromosome. PosG, genetic position (cM); PosP, physical position (Mb; B73v4); Interval, Bayes support interval on the physical map; Var, additive variance explained by the QTL in the multi-QTL model; Analysis, the analyses in which the QTL was identified. Positions and intervals are based on the analysis given the strongest support.

### Identification of QTL linked to flowering time variation

We detected a combined total of 13 QTL across the 4 analyses for the flowering traits *days to anthesis*, *days to silking*, and *anthesis-silking interval* (Table 2). Clusters of flowering QTL were found on both chromosomes 8 and 6 (Table 2). The QTL qDTA8b, qDTS8, and qASI8 were consistently detected across all 4 QTL analyses. Large-effect flowering time QTL have previously been reported on chromosome 8 in both linkage mapping experiments and genome-wide association studies (Jiang et al. 1999; Chardon et al. 2004; Buckler et al. 2009; Coles et al. 2010; Bouchet et al. 2013; Xu et al. 2017; Guo et al. 2018). The flowering QTL we detected on chromosome 8 are in the vicinity of the well-characterized flowering loci *Vgt1* and *Zcn8*. The locus *Vgt1* corresponds to a noncoding region of ∼2 kb that regulates *ZmRap2.7*, an *APETALA-2* like gene located ∼70-kb downstream (Salvi et al. 2007); *Zcn8* is the florigen gene of maize and has a central role in mediating flowering (Meng et al. 2011; Guo et al. 2018). Polymorphisms in *Vgt1* and *Zcn8* have previously been associated with flowering time variation associated with both adaptation to latitude and altitude (Salvi et al. 2007; Ducrocq et al. 2008; Buckler et al. 2009; Romero Navarro et al. 2017; Guo et al. 2018). Given their close proximity, it was not possible to confidently separate the potential effects of *Vgt1* and *Zcn8* in our population, and we consider it possible that the combined effect of variation in both these two loci underlies our QTL on chromosome 8. Mirroring the difference between parents, the PT allele accelerates flowering at qDTA8b, qDTS8, qDTA6 and qDTS6, qDTA1, qDTA7, qDTA8a, qDTS1 and qDTS7 and reduces the *anthesis-silking interval* at qASI1, qASI2, and qASI3 (Supplementary Material).

### Identification of QTL linked to characteristic tassel and sheath traits

PT displays a number of putatively adaptive morphological traits that are characteristic of the Mexican highland group as a whole (Fig. 1a; Eagles and Lothrop 1994; Gonzalez-Segovia et al. 2019). To gain insight into the targets and mechanism of selection during local adaptation, we collected data on characteristic tassel (male inflorescence) morphology, sheath pigmentation, and sheath pubescence traits in our BC_1_S_5_ families (Table 1).

**Fig. 4.**
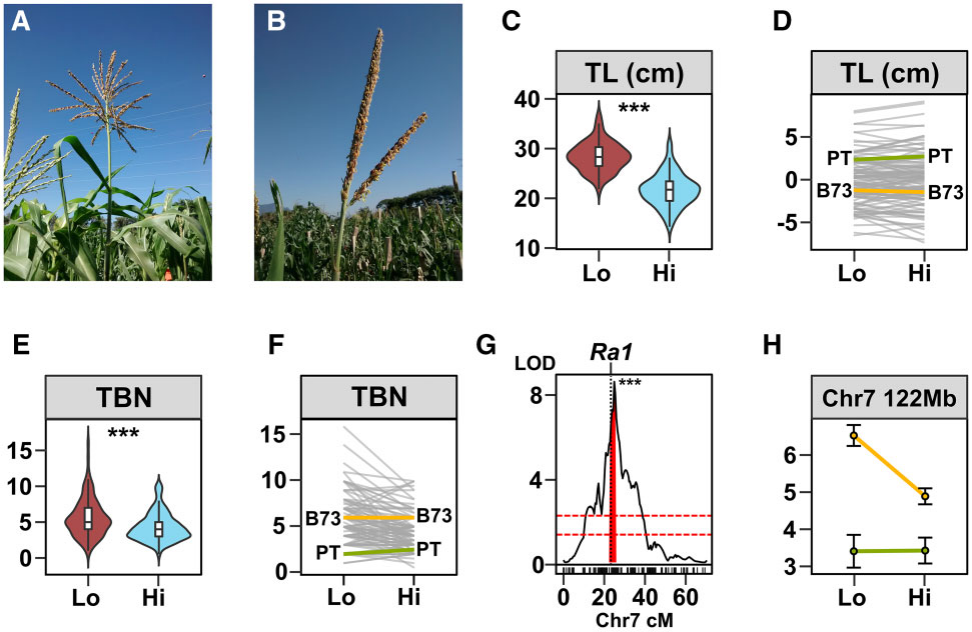
A major QTL for tassel branch number colocalizes with the *Ramosa1* gene. In comparison with typical maize varieties a), tassel branching is strongly reduced in Mexican highland maize b). c) Distribution of TL (cm) in low (Lo) and high (Hi) field sites. Boxes represent the interquartile range with the horizontal line representing the median and whiskers representing 1.5 times the interquartile range. The shape of the violin plot represents probability density of data at different values along the *y*-axis. Support for an environmental main effect shown as *P < 0.05; **P < 0.01; ***P < 0.001. d) Reaction norm plot for *tassel length*. Values shown are G + GEI deviations from the field site average. Line segments connect values for each RIL genotype in the 2 field sites. B73 (yellow) and PT (green) parental values are shown. e, f) as c) and d) for *tassel branch number* (TBN). For f), the plot shows the median for each genotype in each field. g) LOD support (multQTL model, G main effect) for a qTBN7 that colocalizes with the *Ramosa1* (*Ra1*) candidate gene. Red shading indicates a drop 2 LOD interval around the peak marker. Upper and lower dashed red lines show theoretical thresholds for inclusion in the multi-QTL model at α = 0.001 or 0.01, respectively. h) Effect of the chromosome 7 TBN QTL showing trait values for families carrying B73 (yellow) or PT (green) alleles in lowland (Lo) or highland (Hi) field sites.

The PT tassel is large but unbranched with respect to B73 or typical Mexican lowland landraces (Fig. 4, a and b). Across the BC_1_S_5_ population, *tassellength* and *tassel branch number* showed a mild reduction in the highlands compared with the lowland environment (Fig. 4, c–f and Table 1). We identified two QTL linked to t*assel length* (qTL1 and qTL2) and two linked to *tassel branch number* (qTBN2 and qTBN7; Table 2). In common with observations at the whole genotype level, QTL effects for tassel traits were constant in the two environments, and there was no indication of rank-changing QEI (Fig. 4h). For qTL1 and qTL2, the effect was not aligned with the parental difference, with the PT allele being linked to shorter tassels. For *tassel branch number*, the PT allele at qTBN7 was linked to fewer branches, while the PT allele at qTBN2 was linked to a greater number of branches (Fig. 4h). The largest effect was associated with qTBN7 (Fig. 4, g and h) that colocalized with a previously reported tassel branching QTL (Xu et al. 2017; Gonzalez-Segovia et al. 2019) and the *Ramosa1* (*Ra1*, Zm00001d020430, chromosome 7 at 113.57 Mb) candidate gene (Sigmon and Vollbrecht 2010).

**Fig. 5.**
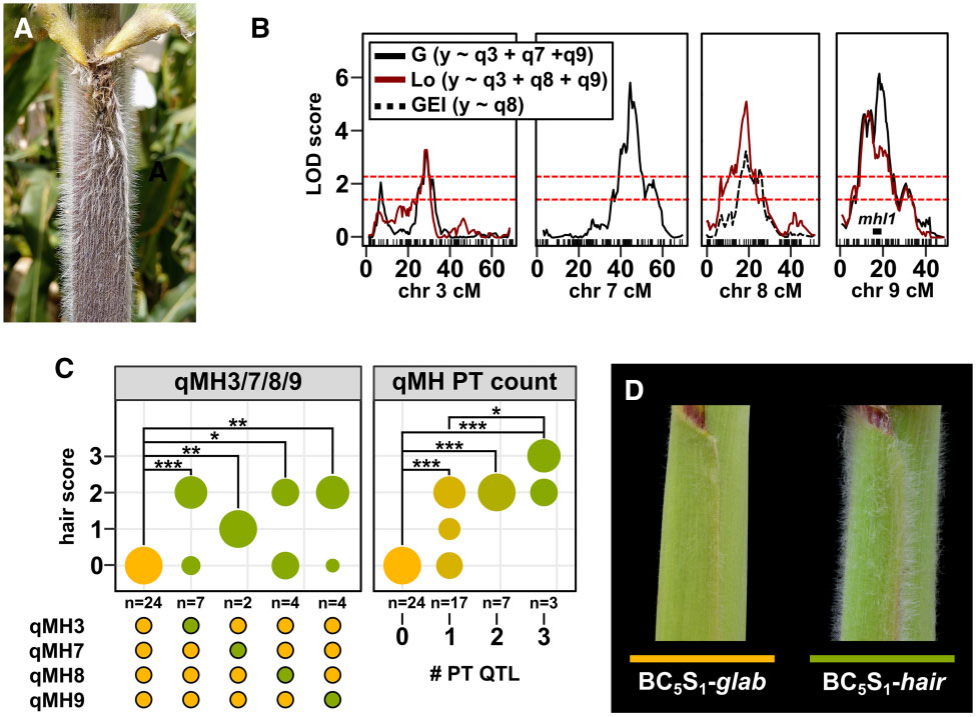
Leaf sheath pubescence is promoted by multiple QTL. a) Mexican highland maize is characterized by extensive leaf sheath pubescence. b) QTLs linked to *macrohair score* (MH) on chromosomes (chr) 3, 7, 8, and 9. Trace shows LOD support in the lowland field (Lo), the genotype main effect (G) or GEI (G×E) analyses. Teosinte introgression on chromosome 9 reported by Hufford et al. (2013) that includes the *mhl1* locus is marked by a black bar. Upper and lower dashed red lines show theoretical thresholds for inclusion in the multi-QTL model at α = 0.001 or 0.01, respectively. c) QTL effect shown as the proportion (shown by circle diameter in the main plot) of RILs scored for different hair score values in a given genotypic class (B73 allele, yellow; PT allele, green). Panels show the effect of allele substitution at the stated QTL in the subset of RILs for which the other QTLs are fixed as B73 and the cumulative effect of increasing the number of PT alleles at qMH 3, 7, 8, or 9. Points below the panel indicate QTL genotype. Fisher’s exact test for pairwise differences between genotype classes, *P < 0.05; **P < 0.01; ***P < 0.001. d) Glabrous (*glab*) and pubescent (*hair*) near-isogenic siblings generated by selection for pubescent plants through 5 generations of backcrossing of a Mexican Conico highland landrace to B73.

PT displays strong leaf sheath pigmentation in comparison to the nonpigmented sheath of B73 (Figs. 1a and 5a). We detected 2 QTL for *pigment intensity* (qINT2 and qINT10) consistently in both environments with no evidence of QEI (Table 2). The qINT2 interval colocalizes with a QTL previously reported in a PT×lowland landrace F_2_ mapping population (Gonzalez-Segovia et al. 2019). Pigment QTL were linked to the well-characterized basic helix–loop–helix (bHLH) regulators of anthocyanin biosynthesis *B1* (Zm00001d000236 chromosome 2, 198.2 Mb) and *R1* (Dooner and Kermicle 1976; Radicella et al. 1992; Selinger et al. 1998; Selinger and Chandler 1999, 2001; Chatham and Juvik 2021; Zm00001d026147, chromosome 10 139.78 Mb).

PT, in common with other Mexican highland landraces, exhibits pronounced leaf sheath pubescence (Fig. 5a). Although macrohairs were present on the leaf sheath in many of the BC_1_S_5_ families, no single family reached the level of pubescence seen in the PT parent, suggesting a complex genetic architecture. Furthermore, the reduced vigor of the BC_1_S_5_ families in the highland location was associated with poor expression of the pubescence trait and difficulty in scoring. Using a semiquantitative scale for evaluation, we identified 4 QTL linked to leaf sheath pubescence (Fig. 5b and Table 2). The QTL interval qMH9 included the *macrohairless1* (*mhl1*) locus that has previously been linked to the production of leaf blade macrohairs in temperate inbred maize (Moose et al. 2004). The qMH9 region also coincided with a previously reported region of introgression from the highland teosinte *Zea mays* ssp. *mexicana* (itself typically pubescent, hereafter *mexicana*) to Mexican highland maize (Hufford et al. 2013; Gonzalez-Segovia et al. 2019; Calfee et al. 2021). This region has been characterized as a chromosomal inversion of ∼3 Mb that displays patterns of selection in highland maize populations (Calfee et al. 2021). The qMH9 interval was relatively large (∼12 cM, estimated to cover ∼100 Mb) and inspection of the LOD profile suggested the possible presence of 2 peaks (Fig. 5b). Although presented here as a single QTL, there may in fact be 2 linked factors.

For all macrohair QTL, the PT allele was associated with greater leaf sheath pubescence. We previously reported difficulty in mapping sheath macrohairs in a PT×lowland landrace F_2_ population because nearly all plants were scored as pubescent in a simple qualitative evaluation (Gonzalez-Segovia et al. 2019). We interpreted this previous observation to indicate the action of several partially dominant factors, each individually sufficient to trigger the production of leaf sheath macrohairs. To further test this hypothesis, we extracted the effect of the PT allele at each macrohair QTL in turn, fixing the other loci as B73 (Fig. 5c). Consistent with genetic redundancy, the PT allele at any macrohair QTL was sufficient to promote a degree of leaf sheath pubescence (Fisher’s exact test; *P* < 0.05 for all four QTL compared with families carrying B73 alleles at all qMH loci; Fig. 5c). Although limited by the size of our population and the qualitative nature of our phenotyping, we could detect a significant difference between families carrying PT alleles at any three macrohair loci and those carrying the PT allele at only one of the loci (Fisher’s exact test; *P* = 0.26; Fig. 5d). Unfortunately, no family carried PT alleles at all four of the loci (this is not unexpected in a BC_1_ population of 98 families). Although the four macrohair loci were individually sufficient to induce leaf sheath macrohair production, we hypothesize that their combined effect (and potentially that of additional loci) is necessary to approach the levels of pubescence of the PT parent.

In parallel with generation of the BC_1_S_5_ population, we also produced pubescent near isogenic lines (NILs) by phenotypic selection and recurrent backcrossing to B73. Here, we initially used several different Mexican highland landrace donors. Material generated from the PT relative Conico (accession Michoacan 21) consistently showed the greatest pubescence and was prioritized for backcrossing and genotypic analysis. A BC_5_S_1_ family showed 3:1 segregation of pubescent to glabrous plants, indicating the action of a single, dominant locus (we did not attempt to distinguish degrees of pubescence in this evaluation, and we do not exclude partial dominance or an additive effect). We selected two strongly pubescent and three strongly glabrous individuals for genotyping using the DArT-Seq platform. The pubescent individuals carried a large block of Mi21 introgression across chromosome 3 that was absent from glabrous plants (Supplementary Fig. 5). Introgression carried in the pubescent NIL spanned the qMH3 interval identified in the B73×PT population, providing an independent line of evidence for a QTL in this location. There was no evidence of significant Mi21 introgression on chromosome 7, 8, or 9 in these BC_5_S_1_ individuals, supporting our previous conclusion that macrohair QTL are individually sufficient to promote a degree of leaf sheath macrohair production. The BC_5_S_1_ family provides a good starting point toward fine mapping and cloning of qMH3.

### Comparison of B73×PT QTL and broader landrace diversity

**Fig. 6.**
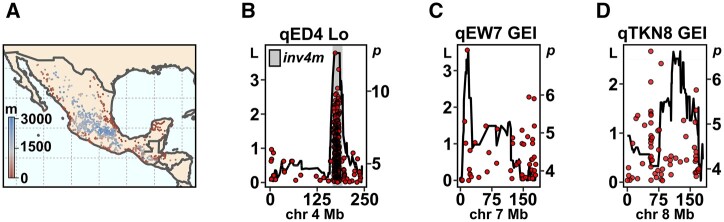
Colocalization of QTL with SNPs showing elevational variation in Mexican landrace maize. a) Geographic distribution of Mexican maize landraces. The color gradient represents elevation of the associated sampling location of maize landrace accessions. b) Support (LOD, L) for an *ear diameter* QTL across chromosome 4 (physical distance) and SNPs (red points) significantly (−log_10_*P*, *P*) associated with elevation in Mexican maize landraces. LOD profile drawn using physically anchored genetic markers and trait values from the lowland (Lo) site. The gray rectangle indicates the position of the previously characterized *inv4m* inversion polymorphism. c, d) as b), showing support for *ear weight* and *total kernel number* QTL on chromosomes 7 and 8, respectively. LOD profiles associated with trait GEI values.

To compare QTL detected in our B73×PT BC_1_S_5_ population to the broader diversity present in Mexican highland maize, we performed an eGWAS using a previously genotyped panel of 1,830 geo-referenced Mexican landrace accessions (Romero Navarro et al. 2017; Gates et al. 2019; Fig. 6a and Supplementary Fig. 6). We selected the top 1,000 SNPs most significantly associated with elevation and compared their physical location with the location of our QTL. The strongest environmental association was detected on chromosome 4 at the previously reported inversion polymorphism *Inv4m* (Romero Navarro et al. 2017; Crow et al. 2020). Although the *ear diamater* QTL qED4 colocalized with this region on chromosome 4 (Fig. 6b), we found no signal linking *Inv4m* to additional yield components or flowering time in our QTL analysis. Further high confidence SNPs were found on chromosomes 2, 3, 5, 7, 8, and 10 (Supplementary Fig. 6 and Supplementary Material). A high confidence SNP on chromosome 7 (17,794,242 Mb on B73v4) was identified adjacent to the GEI peak associated with the *ear weight* QTL qEW7 (Fig. 6c). This region was also identified in a previous experiment to map yield and harvest index QTL in a comparison of lowland and highland Mexican maize (Jiang et al. 1999). In other cases, for example, the *total kernel weight* QTL qTKW8, there was no strong correspondence between the eGWAS hits and the location of the QTL (Fig. 6d). Although we would not draw strong conclusions from differences between eGWAS and QTL results, those cases where they do overlap provide compelling candidates for functional study. For example, the aforementioned SNP on chromosome 7 falls within a gene (Zm00001d019117) encoding a putative transmembrane protein that has been shown in temperate maize to be differentially expressed in response to salt, cold, and UV (Makarevitch et al. 2015).

## Discussion

Evaluation of a B73×PT mapping population in low and high elevation field sites identified QTL associated with both morphological and yield component traits. Although showing plasticity, the genetic architecture of morphological traits was conserved across environments and we saw little evidence of strong rank-changing QEI. Indeed, characteristic highland traits such as pigmentation and pubescence were actually easier to evaluate in the lowland field as a result of the overall greater vigor of the plants. In contrast, we saw greater evidence of QEI (conditional QTL) with respect to yield components, with individual BC_1_S_5_ genotypes mirroring the signature of local adaptation seen in the B73 and PT parents. This broad trend of greater stability of morphological traits compared with yield components is consistent with a previous study mapping maize adaptation across four elevations (Jiang et al. 1999).

In total, across our four analyses, we detected 44 distinct QTLs, 18 of which presented significant QEI based on conditional neutrality. In a previous review of genetic architecture in 37 studies, the authors estimated that ∼60% of the QTLs detected displayed QEI, but that there was only evidence for antagonistic pleiotropy in ∼2% of cases (Des Marais et al. 2013). This broad trend was reflected in a recent multisite mapping experiment in switchgrass (*Panicum virgatum* L.) in which the majority of QTL associated with adaptive traits showed conditional positive effects in their home environment with little or no detectable effect or cost in other environments (Lowry et al. 2019). Detecting antagonistic pleiotropy requires higher statistical power than identification of conditional effects (furthermore, the latter can be supported by a failure to detect an effect in certain environments), resulting in a potential bias in the classification of QEI (Anderson et al. 2011). In our study, the statistical power necessary to dissect QEI is limited by the size of the mapping population and the number of trials and locations evaluated. Furthermore, as a result of extensive management employed at the lowland site, plants would have been exposed to few of the stresses traditionally faced in tropical lowland fields. Any buffering of the potential costs of highland variants in the lowland site would push the genetic architecture from antagonistic pleiotropy toward conditionality. Similarly, by not employing traditional highland management practices (deep planting, absence of supplemental irrigation) the deleterious effects of lowland adapted alleles might also be underestimated.

The flowering QTL qDTA8, qDTS8, and qASI8 overlap a ∼10-Mb region that contains the two well-characterized flowering genes *ZmRap2.7* and *Zcn8*. This region and/or these genes have been reproducibly detected in linkage- and association-mapping studies of maize flowering time (Chardon et al. 2004; Buckler et al. 2009; Steinhoff et al. 2012; Li et al. 2016; Romero Navarro et al. 2017), temperate adaptation (Ducrocq et al. 2008; Bouchet et al. 2013; Guo et al. 2018; Castelletti et al. 2020), and adaptation to the Mexican Highlands (Gates et al. 2019; Janzen et al. 2021; Wang et al. 2021). An early flowering *Vgt1* allele from northern germplasm has previously been associated with a miniature transposon (MITE) insertion upstream of *ZmRap2.7*, although the absence of the MITE alone did not explain late flowering *Vgt1* alleles (Buckler et al. 2009). In a *Zcn8* association study using maize and teosinte, the haplotype associated with earliest flowering (A-Del) was hypothesized to have originated in highland teosinte *mexicana* and to have been transferred to cultivated maize by introgression (Guo et al. 2018). Interestingly, in this same study the authors report PT to carry both the MITE-associated allele of *Vgt1* and the A-Del haplotype of *Zcn8*. Although we have not sequenced the *Vgt1* and *Zcn8* alleles present in our mapping population and available genome sequence data do not provide good coverage in this region, our linkage mapping results are consistent with this previous association analysis.

The flowering time QTL qDTA6 and qDTS6 are in close proximity to the gene *Peamt2* (Zm00001eb294690, chromosome 6 ∼166.5 Mb), an ortholog of the *Arabidopsis XIPOTL1* gene encoding for a phosphoethanolamine N-methyltransferase (PEAMT). PEAMT catalyzes the transformation of phosphocholine to phosphatidylcholine (PC; Cruz-Ramírez et al. 2004; Sánchez Martínez 2018). The balance between PC and its precursors has previously been associated with flowering time regulation in *Arabidopsis* (Nakamura et al. 2014) and implicated in early flowering in Mexican highland maize (Rodríguez-Zapata et al. 2021). Fine mapping and metabolic studies would be needed to confirm the possible role of variation of *Peamt2* in maize flowering time variation.

We identified several morphological QTL that could be confidently associated with strong candidate genes. For sheath *pigment intensity*, qPINT2 and qPINT10 colocalize with the loci *B1* and *R1*, respectively. The qPINT2 locus had the greatest effect of the two (explaining ∼40% variance) with the PT allele promoting pigmentation. The *B1* gene encodes a bHLH transcription factor that regulates the temporal and tissue-specific expression of genes that produce anthocyanins (Ludwig et al. 1989; Petroni et al. 2000; Chatham and Juvik 2021). Interestingly, *B1* was also identified in a mapping cross between lowland and highland teosinte, the latter showing the leaf sheath pigmentation also seen in highland maize (Lauter et al. 2004). Several independently derived *B1* alleles have been linked to leaf sheath pigmentation (Dooner and Kermicle 1976; Radicella et al. 1992; Selinger et al. 1998; Selinger and Chandler 1999, 2001; Chatham and Juvik 2021), indicating the ready production of functional diversity at this locus and implicating convergent selection (Stern 2013) for pigmentation among highland *Zea*, supporting an adaptive role (Doebley 1984; Lauter et al. 2004). In reported cases, functional variation at *B1* is driven by patterns of upstream transposon insertion that impact gene expression. For example, *B-Bolivia* induces the biosynthesis of anthocyanin in both vegetative tissue and the aleurone of the grain, while the *B-Mex7* allele, which was identified from the Mexican highland landrace Cacahuazintle, induces pigment in the margins of the leaf sheath (Chandler et al. 1989; Radicella et al. 1992; Selinger and Chandler 1999). Further sequencing of *B1* alleles from highland maize will shed greater light on patterns of diversity and the origin of different alleles. Leaf sheath pigmentation, unlike sheath pubescence, is shared with South American highland maize (Janzen et al. 2021). Dark red pigmentation in the sheath can help the plant to absorb more solar radiation and keep the plant warmer in a cold environment and might also protect DNA from damage due to higher UV-B radiation in the highlands (Barthakur 1974; Eagles and Lothrop 1994; Casati and Walbot 2005)—although it is unclear why such protection might be required more so in the sheath than in the leaf blades.

We identified QTL associated with leaf sheath pubescence on chromosomes 3, 7, 8, and 9. Our QTL on chromosome 9 is consistent with previous observations colocalizing (1) a leaf blade macrohair mutation in temperate maize (Moose et al. 2004); (2) a leaf sheath pubescence QTL in *mexicana* teosinte (Lauter et al. 2004); (3) introgression from *mexicana* to highland maize (Hufford et al. 2013; Gonzalez-Segovia et al. 2019); and (4) a ∼3-Mb inversion that displays a pattern of clinal selection across elevation in Mexican maize (Calfee et al. 2021). Identification of a PT allele linked to leaf sheath pubescence in this same region of chromosome 9 adds further support to the hypothesis of adaptive introgression at this locus (Wilkes 1972; Gonzalez-Segovia et al. 2019). In this context, it is interesting to note that all macrohair QTL identified appeared to be sufficient on their own to induce a degree of leaf sheath pubescence, although their combined action would likely be needed to approach the level of pubescence of the PT parent. Limitations of population size and the semiquantitative nature of our evaluation prevent strong conclusions concerning additivity or interactions among macrohair QTL. Nonetheless, our data suggest that qMH9 is just one of a number of contributing loci, the origin of the trait being a complex mix of wild-relative introgression and *de novo* mutation. Fine mapping and molecular cloning of the genes underlying macrohair QTL would allow a far more detailed view of the history of leaf sheath pubescence and associated genetic variants in Mexican highland maize. Pubescence extends the boundary layer around the stem and could act as protection from cold by preventing heat loss or conserve water by minimizing transpiration (Chalker-Scott 1999). In addition, leaf pubescence has been suggested to aid in the capture of dew and, as a consequence, increasing water use efficiency in the plant (Konrad et al. 2015), a potentially important trait during the initial dry months of the highland growth cycle, prior to the beginning of the rainy season. We did not observe any strong correlation between pubescence and yield components. However, further experiments making use of the range of pubescence in our inbred families or derived NILs, might have the power to detect more subtle effects in either controlled conditions or highland trials.

The rich diversity of Mexican landrace maize is closely tied to local adaptation. Yet, this same specialization places these varieties at risk from future climate change (Mercer and Perales 2010; Bellon et al. 2011; Romero et al. 2020). In a study to project landrace distribution under different climate change scenarios, PT was identified as the most vulnerable landrace (Ureta et al. 2012), although, as the authors note, models based on current distribution and climate do not take into account the full range of environmental, biotic, and cultural factors that impact diversity and distribution. In the specific case of PT, limited yield potential in comparison to more modern landraces is likely to see it abandoned by farmers (https://www.biodiversidad.gob.mx/diversidad/proyectoMaices). That said, PT has contributed to the broader Mexican highland group (Reif et al. 2006; Warburton et al. 2008; Arteaga et al. 2016) and locally adapted alleles will likely be conserved. The fate of the Mexican highland maize group will be influenced by its ability to adapt to climate change. PT and PT allele effects were largely stable and GEI was driven by plasticity associated with B73. As such, our data would support cautious optimism that highland varieties might maintain current levels of productivity in the face of future climate change. However, if climate change results in the expansion of lower elevation varieties to the highlands, the home site advantage of traditional highland landraces might be eroded. Ultimately, the conservation of maize diversity, along with the responsible and equitable use of this unique resource, will be informed by a greater understanding of the physiological and mechanistic basis of local adaptation. With an increase in mapping resolution (for example, by using larger or more diverse populations) and the availability of high-quality landrace genome assemblies, it will be possible to take important further steps toward defining not only the genetic architecture but also the genes and genetic variants that underlie local adaptation in maize landraces.

## Germplasm and data availability

*Germplasm:* Seed of PT (CIMMYTMA 2233) and Mi21 (CIMMYTMA 1872) are available from the CIMMyT Germplasm Bank https://www.cimmyt.org/resources/seed-request/. All other materials are available from the corresponding author subject to SMTA compliance (https://mls.planttreaty.org/itt/index.php), costs of propagation, and export if outside of Mexico. The following data sets can be found online on Figshare: https://figshare.com/articles/dataset/B73xPT_QTL/16608517. *Phenotypic data*: BLUPs and fitted values estimated for diverse traits for 98 B73×PT BC_1_S_5_ families. *Genetic map*: genetic map of the B73×PT BC_1_S_5_ mapping population. *QTL LOD profile*: LOD profile of the multi-QTL models for different traits for each set of phenotypic data. *Effect Plots*: estimated effect of the QTLs detected for a set of phenotypic data using fitted values. *Reaction norms*: contains reaction norms estimated for all the QTLs detected in Lo and Hi phenotypic sets using fitted values. *Elevation eGWAS*: results of the eGWAS analysis.

Additonal supplemental figures and supplemental information referenced in the text are available at G3 online.

## Supplementary Material

jkab447_Supplementary_Figure_S1

jkab447_Supplementary_Figure_S2

jkab447_Supplementary_Figure_S3

jkab447_Supplementary_Figure_S4

jkab447_Supplementary_Figure_S5

jkab447_Supplementary_Figure_S6

jkab447_Supplemental_Material

jkab447_Supplemental_Information

jkab447_Supplemental_Material
