# Supplementary material for: A B73×Palomero Toluqueño mapping population reveals local adaptation in Mexican highland maize
Source: G3 (Bethesda). 2022 Jan 3;12(3):jkab447. doi: 10.1093/g3journal/jkab447 (PMC8896015; doi:10.1093/g3journal/jkab447)
Supplement: jkab447_Supplementary_Figure_S1 [file jkab447_supplementary_figure_s1.pdf]

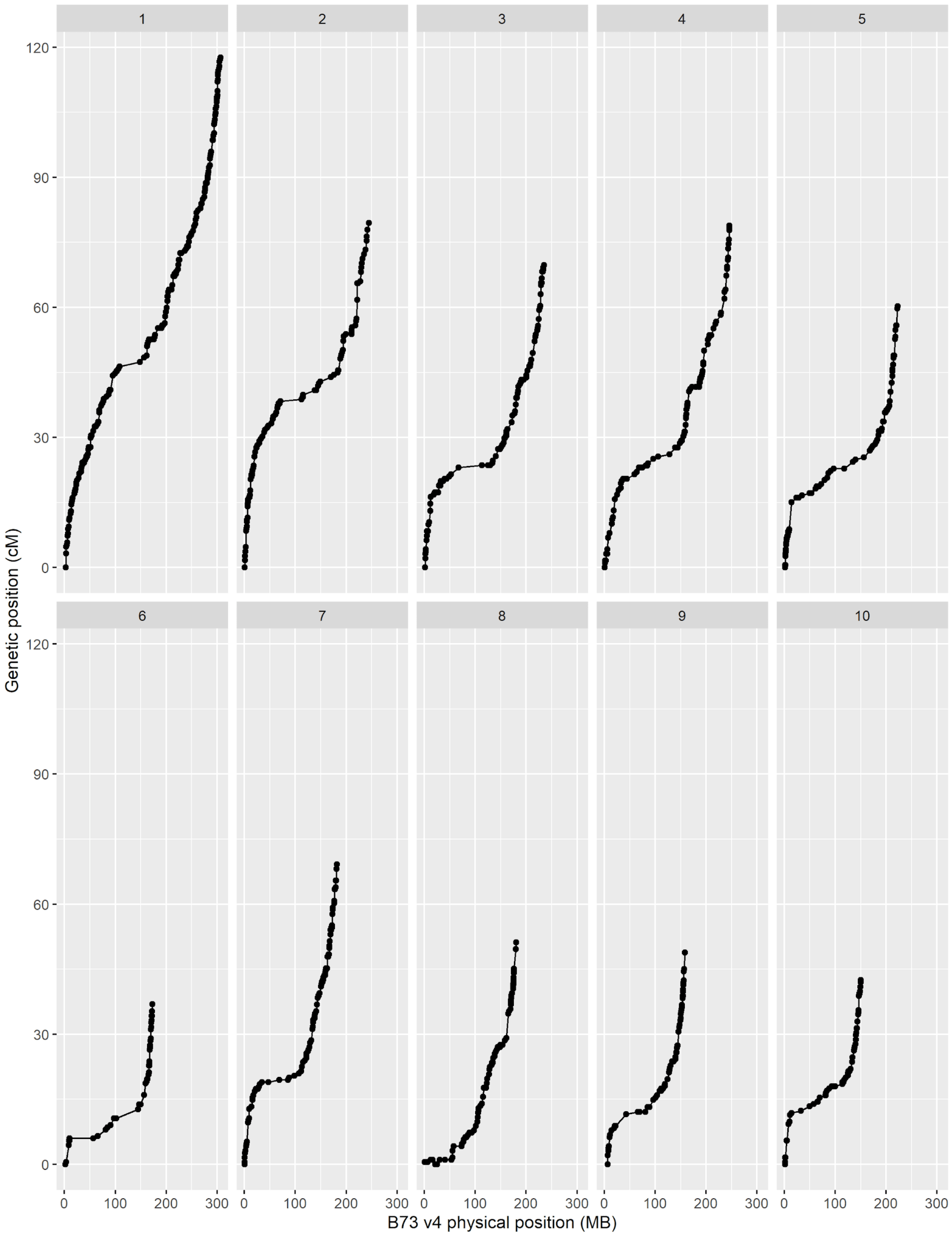

Figure S1. Physical position of a marker with respect to the v4 B73 reference genome (x axis) and the estimated genetic position (y axis). Each dot represents a marker of the genetic map of the B73 x PT BC_1S_5 population.
