# Supplementary material for: A B73×Palomero Toluqueño mapping population reveals local adaptation in Mexican highland maize
Source: G3 (Bethesda). 2022 Jan 3;12(3):jkab447. doi: 10.1093/g3journal/jkab447 (PMC8896015; doi:10.1093/g3journal/jkab447)
Supplement: jkab447_Supplementary_Figure_S2 [file jkab447_supplementary_figure_s2.pdf]

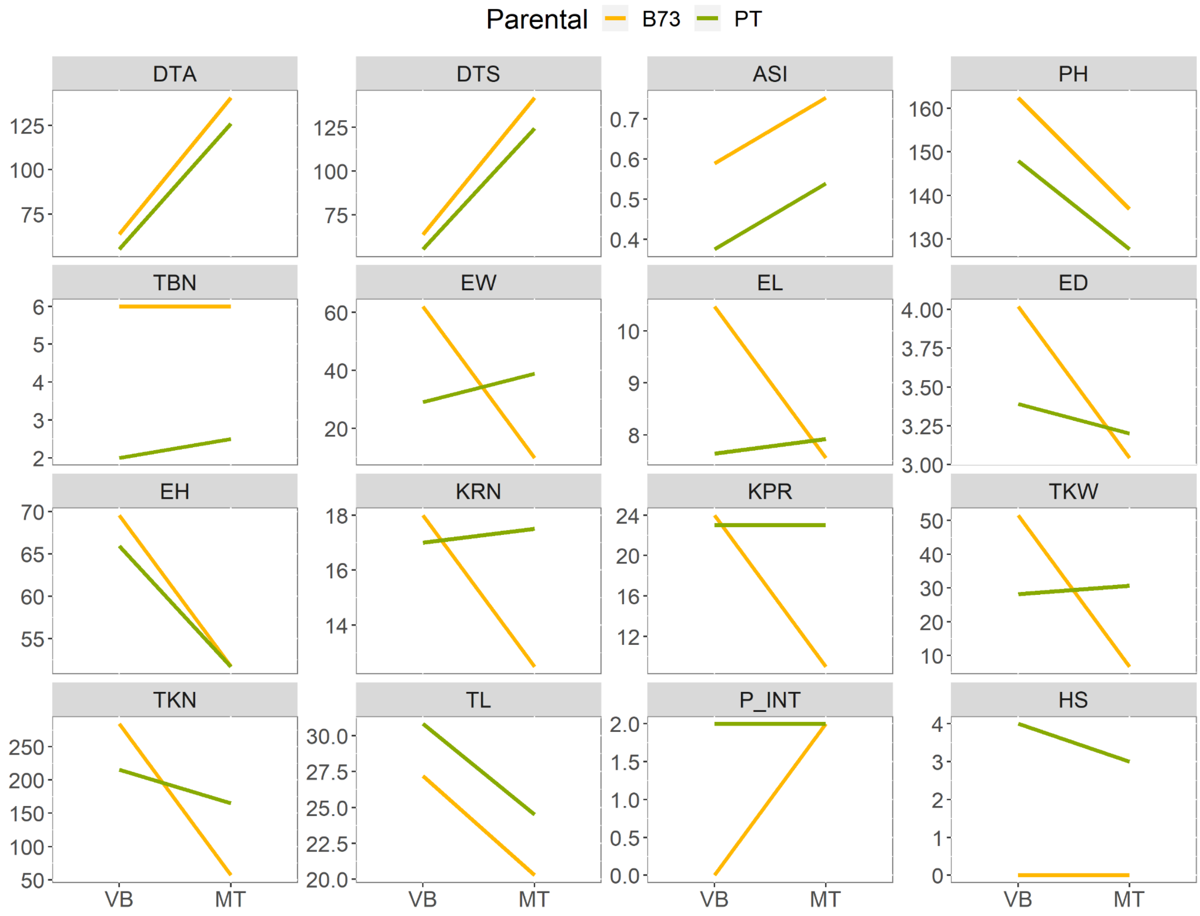

Figure S2. Reaction norms of B73 (green line) and Palomero Toluqueño landrace (yellow line) grown in lowland (VB) and highland (MT) field sites. Trait descriptions shown in main text Table 1. The fitted values were estimated by adding BLUPs of the G+GEI to the estimated location term.
