# Supplementary material for: A B73×Palomero Toluqueño mapping population reveals local adaptation in Mexican highland maize
Source: G3 (Bethesda). 2022 Jan 3;12(3):jkab447. doi: 10.1093/g3journal/jkab447 (PMC8896015; doi:10.1093/g3journal/jkab447)
Supplement: jkab447_Supplementary_Figure_S3 [file jkab447_supplementary_figure_s3.pdf]

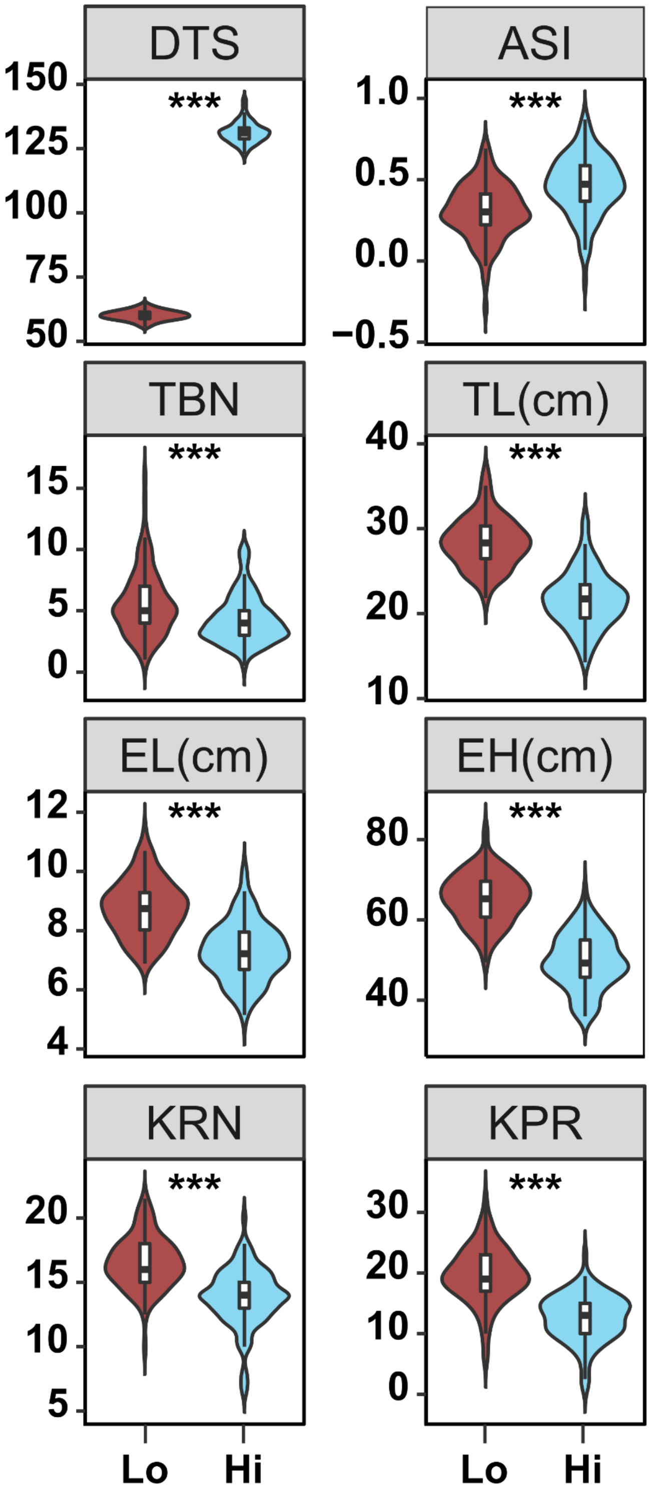

Figure S3. Distribution of phenotypic traits for B73xPT recombinant inbred lines grown in lowland (Lo) or highland (Hi) field sites. Trait codes and descriptions shown in main text Table 1. Fitted values for each genotype/location were generated by adding G and GEI BLUPs to the estimated location term. Violin plots for the count traits TBN, KPR, KRN were generated using genotype medians for each location. Boxes represent the interquartile range with the horizontal line representing the median and whiskers representing 1.5 times the interquartile ranges. The shape of the violin plot represents probability density at different values along the y-axis. Wilcoxon adjusted P values for an environmental effect shown as *: $p < 0.05$; **: $p < 0.001$; ***: $p < 0.0001$.
