# Supplementary material for: A B73×Palomero Toluqueño mapping population reveals local adaptation in Mexican highland maize
Source: G3 (Bethesda). 2022 Jan 3;12(3):jkab447. doi: 10.1093/g3journal/jkab447 (PMC8896015; doi:10.1093/g3journal/jkab447)
Supplement: jkab447_Supplementary_Figure_S4 [file jkab447_supplementary_figure_s4.pdf]

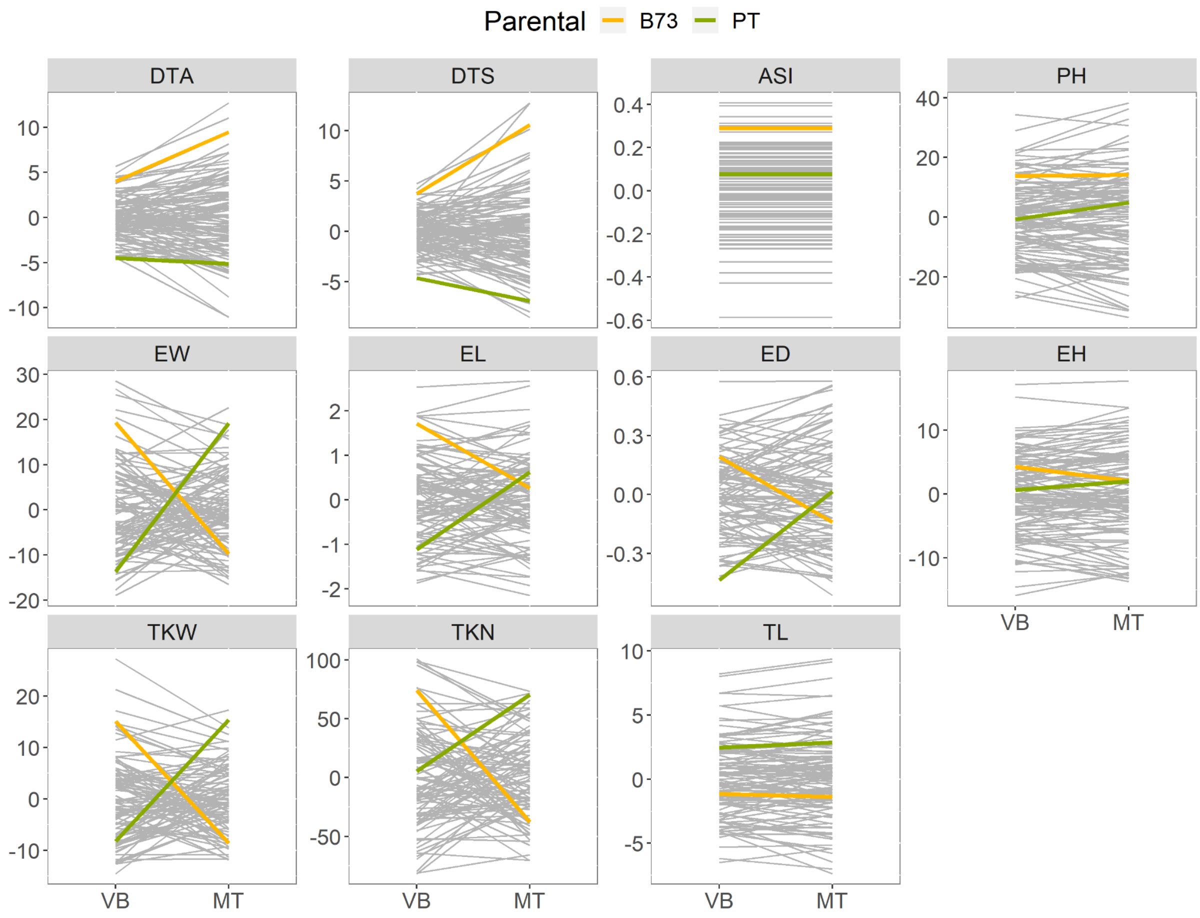

Figure S4. Reaction norm plots of phenotypic traits for B73xPT recombinant inbred lines grown in lowland (VB) or highland (MT) field sites. Trait descriptions were shown in main text Table 1. Values shown are the sum of BLUPs for G and GEI terms for each genotype. Gray line segments connect values for each RIL genotype in the two field sites. Parental values are shown in thick yellow (B73) and green (PT) lines.
