# Supplementary material for: A B73×Palomero Toluqueño mapping population reveals local adaptation in Mexican highland maize
Source: G3 (Bethesda). 2022 Jan 3;12(3):jkab447. doi: 10.1093/g3journal/jkab447 (PMC8896015; doi:10.1093/g3journal/jkab447)
Supplement: jkab447_Supplementary_Figure_S5 [file jkab447_supplementary_figure_s5.pdf]

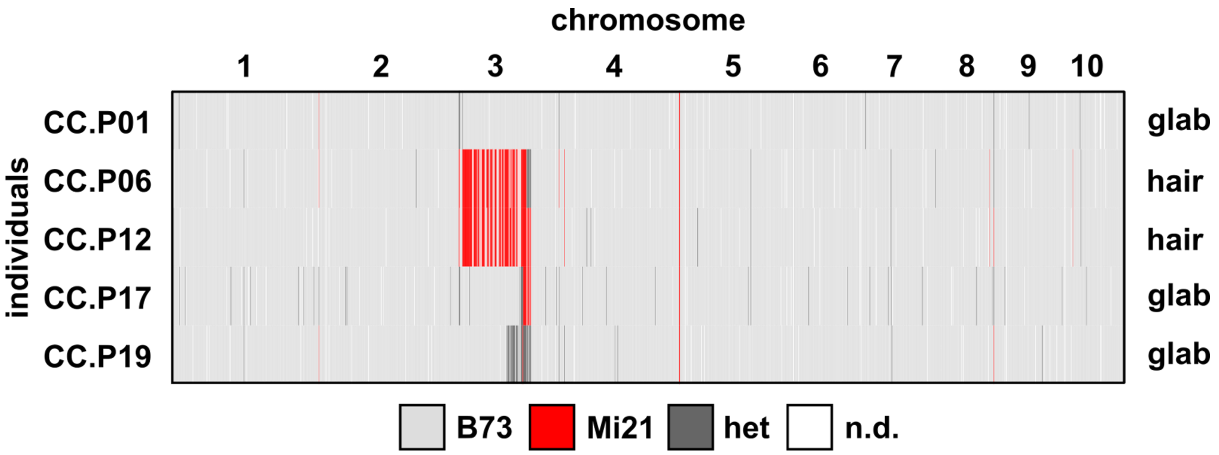

Figure S5. Pubescent segregants from a B73xMi21 BC_5S_1 family contain Mi21 introgression on chromosome 3. Three glabrous (glab) and two pubescent (hair) individuals genotyped with DaRT-SEQ (Edet *et al.*, 2018). A set of 2030 markers were converted to ABH format with reference to B73 or PT (used here as proxy for Mi21) and visualized using R/ABHgenotypeR (Reuscher & Furuta, 2016).
