# Supplementary material for: A B73×Palomero Toluqueño mapping population reveals local adaptation in Mexican highland maize
Source: G3 (Bethesda). 2022 Jan 3;12(3):jkab447. doi: 10.1093/g3journal/jkab447 (PMC8896015; doi:10.1093/g3journal/jkab447)
Supplement: jkab447_Supplementary_Figure_S6 [file jkab447_supplementary_figure_s6.pdf]

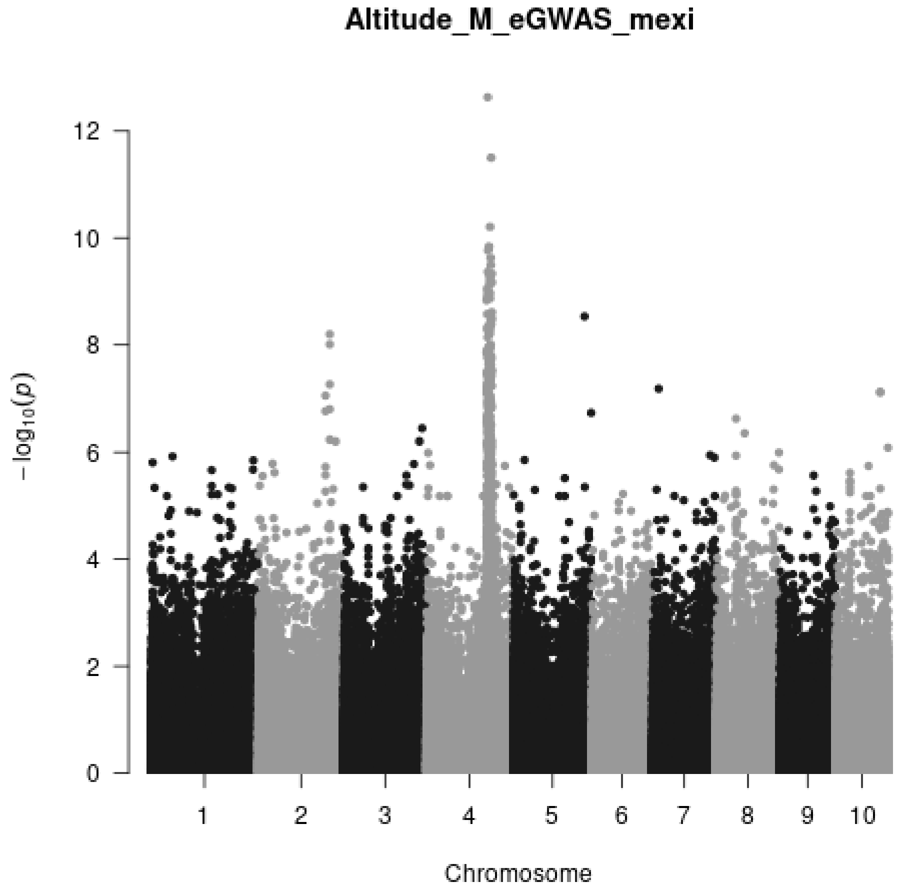

Figure S6. Manhattan plot showing the genome-wide associations for altitude of Mexican maize landraces. The x axis represents the position of maize chromosomes, and the y axis shows the $-\log_{10}(P)$ of each marker.
