# Supplementary material for: A B73×Palomero Toluqueño mapping population reveals local adaptation in Mexican highland maize
Source: G3 (Bethesda). 2022 Jan 3;12(3):jkab447. doi: 10.1093/g3journal/jkab447 (PMC8896015; doi:10.1093/g3journal/jkab447)
Supplement: jkab447_Supplemental_Material [file jkab447_supplemental_material.docx]

**Figure S1. Physical position of a marker with respect to the v4 B73 reference genome (x axis) and the estimated genetic position (y axis).**

**Figure S2.** **Reaction norms of B73 (green line) and Palomero Toluqueño landrace (yellow line) grown in lowland (Lo) and highland (Hi) field sites.**

**Figure S3. Distribution of phenotypic traits for B73xPT recombinant inbred lines grown in lowland (Lo) or highland (Hi) field sites.**

**Figure S4. Reaction norm plots of phenotypic traits for B73xPT recombinant inbred lines grown in lowland (Lo) or highland (Hi) field sites.**

**Figure S5. Pubescent segregants from a B73xMi21 BC_5_S_1_ family contain Mi21 introgression on chromosome 3.**

**Figure S6. Manhattan plot showing the genome-wide associations for altitude of Mexican maize landraces.**
